# Anæsthetics in Labor

**Published:** 1884-03

**Authors:** C. L. Swift

**Affiliations:** Auburn, N. Y.


### ANÆSTHETICS IN LABOR.

Dr. C. L. Swift, Auburn, N. Y.

[*Read before the Cayuga County Medical Society.*]

In every age of the world "man has sought out many inventions." Some of these have remained and are an honor to the inventor. Others have been discarded as "worse than useless."

New discoveries have been made in the scientific world. Old theories have been given up and new ones accepted.

What was once practiced and followed is now cast aside and other methods take their places.

There have been those who have accepted the theories simply because they were new and popular. They have tried to practice what was received with most favor during their stage of action, and have tried as best they could to improve and use the latest inventions, and discoveries, sometimes producing disastrous consequences thereby.

Among these discoveries is one which will ever remain to honor the memory of its discoverers, viz.: *Anæsthetics.*

Ether was discovered and introduced into general practice as an anæsthetic by Drs. Horace Wells, of Hartford, Conn., and T. G. Morton, of Boston, in the year 1846. Chloroform was discovered as an anæsthetic in the following year by Professor Simpson, of Edinburgh.

At first these agents were used with great caution from fear of injurious results, but with more frequent use and experience with them this fear is fast passing away, so that to-day they are used in many forms of pain and disease to which the body is heir, such as toothache, neuralgias, spasms, etc., and with some are becoming favorite remedies in cases of labor.

The use of anæsthetics is a great boon to mankind. They have their place in medicine and surgery, but they should be kept in their place and their use restricted to such cases as only demand them.

Are anæsthetics injurious in labor? What harm may result from their use? Is there anything that can be substituted in controlling or directing the pains of labor? These are questions of vital importance to every one who is practicing medicine.

The general effect of Chloroform on the system is well known to every physician, and the indiscriminate use of it is highly prejudicial to the health of patients in ordinary conditions calling for its use. The using of Ether is attended with less danger than Chloroform, but there are also objections to that.

Dr. Henry M. Lyman, in his work on anæsthetics, says: "The contraindications to the use of anæsthetics in obstetrical practice are cardiac and respiratory paresis, which must positively prohibit their use under all circumstances."

Retardation and diminution of the pains are also reasons for withdrawing the anæsthetic. There is always danger in such cases if the wishes of the patient are gratified to their full extent that the use of the forceps will become necessary in order to complete the labor.

The dangers of an anæsthetic in labor are not only the same as in ordinary use, but other dangers may arise which are aggravated or produced by it.

The theory that parturient patients are not as susceptible to anæsthesia as others is not true.

Dr. Lyman says: "The parturient woman is apparently as promptly overpowered by Chloroform or Ether as the non-parturient."

That death may follow the use of Chloroform the following cases, taken from the work of Dr. Lyman, will show:

" A lady during the early stage of labor in the care of a nurse, her physician being absent, inhaled about five drachms of Chloroform from a handkerchief, went to sleep, and was found dead and cold when the nurse, who had also fallen asleep, awoke.

"Lady six times pregnant had taken Chloroform at each confinement. September 20th, 1858, her pains commenced at two A. M. No one but a nurse in attendance. About twenty minutes to eight A. M. expulsive pains came on, when she called for Chloroform. After breathing it a few times from a handkerchief she threw herself violently back, gave a gasp or two, a slight gurgle was heard in her throat, and respiration and the pulse instantly ceased. Her physician arrived on the spot ten minutes later and found her dead. The quantity used did not exceed two drachms.

"Female twenty-five years, multipera. Labor pains continued during evening and night. The next morning at seven o'clock the membranes ruptured, shoulder presentation. To facilitate version, Chloroform was given by the nurse without calling upon the attending physician. The patient did not arouse after the operation. The physician was called, who found the pulse small, the face cyanotic, the inspirations short and frequent. Patient died in ten minutes.

"Female twenty-two years, primipara. The head of the child was at the point of birth when a slight convulsion occurred. Chloroform was given and the patient was kept under its influence. After the delivery of the head, while the uterus was contracting, the patient shuddered and her pulse ceased—she was dead."

Death to the patient is not the only result to be feared. The fœtus may suffer if the mother does not.

Dr. Lyman, speaking on this subject, says: "The possible effects of Chloroform upon the fœtus must not be neglected. When administered in small and stimulating doses during the pains alone the new-born infant rarely exhibits any symptoms that can be certainly referred to the anæsthetic, but an excessive saturation of the blood may prove dangerous to the child."

One of the dangerous complications of labor is hæmorrhage. Dr. Lyman in speaking of it says: "The possibility of hæmorrhage as a consequence of too abundant or too long continued use of Chloroform should not be overlooked."

Under favorable conditions it is very unpleasant, to say the least, but when it follows the giving of Ether or Chloroform once meeting it will usually satisfy the ordinary practitioner.

You seldom get the regular cannon-ball contractions of the uterus unless you administer Ergot or Ustilago in appreciable doses, and if you do sometimes they are not as firm and you have to watch your patient with a good deal of care for fear of postpartum hæmorrhage.

Are not these reasons sufficient to cause the candid homœopathic physician who has such abundant resources for knowledge to discard their use?

The homœopathic physician, with his knowledge of the power of potentized drugs and a firm belief in the law of similars, has no need of an anæsthetic in cases of labor.

Labor is a normal and physiological process, and when nature works according to the laws which govern it, why interfere with these laws by giving an anæsthetic?

Dr. Lyman says: "In all cases of normal parturition the employment of anæsthetics is as undesirable as would be the practice of using opiates during the period of normal menstruation. The use of them in such labor can afford no advantage—may even work an injury to the patient."

From experience we learn that there are many causes both inside and outside the female economy by which nature's laws are perverted, and, if left to herself alone, she often fails to carry them out. Then should the physician strive to assist and direct her in accomplishing the desired end. He should not do this by giving an anæsthetic, but by carefully examining the case—its history, its complications, and the concomitant symptoms, all taken together—and then select the most similar remedy for the condition. The labor will then terminate much more satisfactory to the patient and friends and give greater credit to the physician in charge.

Many a physician can testify to the effect of Cham. in cases attended with restlessness, thirst; patients are cross, nervous—will get up, think the pains are too severe—last too long; when they scold at everything and nothing suits them.

A bit of Chloroform or Ether would quiet them; so will Cham. Which is safest and best?

Mrs. S. was taken in labor. Pains would reach to a certain point and then cease. There was great suffering. On examination there was great tenderness of osuteri, walls of the vagina, and vulva. The extreme tenderness could have been overcome by an anæsthetic, but the result would not have been as beneficial as it was under Platina, which removed the tenderness and allowed labor to proceed normally.

There are many remedies from which the physician will be called to choose, and he may often fail in producing the desired result; but he must never forget that, in order to benefit the patient, the remedy must be indicated.

There is no such thing as giving Caulophyllum, Cimicifuga, Collinsonia, Pulsatilla, Sepia, or any other remedy, because Dr. So-and-so did, and expect to obtain the best results. But the remedy most similar to the case must be given.

There are cases where remedies will fail and the physician must resort to mechanical means. The forceps must be applied. Surely it is necessary to give an anæsthetic now?

Dr. Guernsey says: "The use of anæsthetics in these operations is particularly objectionable, since their use tends to increase the danger; for when pain is produced by pressure with the forceps, we know all is not right and hasten to correct the error. But when the patient is rendered unconscious by the use of an anæsthetic, this valuable indication is lost." (Vide *Obstetrics*, p. 268, third edition.)

If physicians will spend more time in becoming acquainted with the materia medica and less in looking for something new, they will be more proficient in the art of controlling morbid conditions, and the less will they desire anæsthetics in cases of labor.

